# Air traffic controller work state recognition based on improved xception network

**DOI:** 10.1371/journal.pone.0322404

**Published:** 2025-05-07

**Authors:** Miao Guo, Zheng Guan

**Affiliations:** 1 Airport Management College, Shanghai Civil Aviation College, Shanghai, China; 2 Department of Operational Management, Shenzhen Airlines Co., Ltd., Shenzhen, China; Newcastle University, UNITED KINGDOM OF GREAT BRITAIN AND NORTHERN IRELAND

## Abstract

In the current context of rapid development of air traffic, the long-time and high-intensity working environment can easily lead to controllers’ fatigue state, which in turn affects flight safety. Different from the traditional Mini-Xception pre-training network oriented to the classification task, the study improves it so that it can effectively process multi-dimensional time-series data of air traffic controllers’ facial expressions and emotional changes. On its basis, a dynamic time-series data processing module is introduced and combined with a multi-task learning framework and a technique that combines multi-level feature extraction and emotional state analysis to realize the joint recognition of facial expressions and work states, such as fatigue and stress. The experiment findings denotes that the new model has the highest accuracy of 94.36% in detecting eye fatigue, the highest recall rate of 91.68%, and the maximum area under the curve test value of 93.02%. Compared to similar detection models, its average detection time is shortened by 1.9 seconds, with the highest accuracy of 95% in detecting 180 human eye images and an average fatigue detection of 91%. The innovation of the research is to utilize Mini-Xception network for real-time analysis of dynamic features of facial expressions and correlate them with the actual work performance of the controllers, which proposes a new multi-task learning framework, improves the accuracy and stability of the recognition, and provides a new idea and technical support for intelligent monitoring and control of air traffic management system.

## 1. Introduction

With the continuous increase of air traffic flow, the work pressure of air traffic controllers is also gradually increasing. The complex flight scheduling, constantly changing weather conditions, and response to unexpected situations all require air traffic controllers to maintain a high level of vigilance and decision-making ability in high load and high-intensity work environments [1]. This highly concentrated attention and frequent operations not only increase workload, but also make it easier for controllers to experience fatigue and distraction after long hours of work. Therefore, fatigue has become a critical factor affecting the working status of air traffic controllers. Previous studies denote that fatigue not only weakens the attention and reaction speed of air traffic controllers, but may also lead to a decrease in decision-making ability, thereby increasing the risk of air traffic accidents [2]. Therefore, identifying and detecting the fatigue status of air traffic controllers is of great meaning for ensuring flight safety. However, there are still some key research gaps in the field of existing air traffic controller work state recognition that need to be further explored. First, most of the existing research focuses on the analysis of single modal data, such as working state recognition based on speech signals or physiological signals, and lacks the fusion analysis of multimodal data. The working state of air traffic controllers is affected by a variety of factors, and it is difficult for single modal data to fully reflect their complex working state. Secondly, the existing methods are deficient in real-time and adaptability. The working environment of air traffic controllers changes dynamically, which requires that the recognition model can quickly adapt to different working scenarios and individual differences. To this end, the research achieves accurate recognition of the controller’s working state by improving the Xception network and utilizing deep convolutional neural networks for deep learning of the controller’s multimodal data, such as facial expressions and posture changes. Compared with the existing deep learning-based methods for air traffic controller operating state recognition, the study differs in three ways. First, multimodal data fusion, which combines facial expression, eye fatigue, and posture changes to achieve more comprehensive state recognition; second, dynamic time-series analysis, which improves the stability and accuracy of recognition by processing continuous expression changes through an improved Mini-Xception network; and third, a multi-task learning framework, which detects fatigue, stress, and other states at the same time and optimizes the computational efficiency to be more applicable to the air traffic control working environment. Compared with traditional methods, the innovation of the study is that the multi-level feature extraction capability of the deep learning model can better adapt to the variability and challenges of the working environment of air traffic controllers. The contribution of the research is that it not only provides an efficient and accurate solution for air traffic controllers’ work status recognition, but also provides a technical reference for work status monitoring in other high-load occupations. By filling the technical gaps in multimodal data fusion, real-time optimization and fine-grained identification, the research provides new ideas and methods to improve aviation safety management.

## 2. Related works

Zhang et al. [3] believed that traditional fatigue detection methods either require inconvenient sensor connections or utilize camera systems that are sensitive to light and may leak privacy. To this end, the research team proposed a new driver fatigue detection model by combining single line radar monitoring and signal recovery algorithms. The experimental outcomes denoted that the accuracy of fatigue detection in the time series detection of 20 volunteers using this model could reach 89.47%. To lessen various traffic accidents caused by fatigue, Zhang Z et al. [4] proposed a new system that combines data sampling, deep fatigue feature extraction, and fatigue assessment to detect this adverse state early. The experimental outcomes denoted that the system had high accuracy in detecting driving fatigue. To raise the accuracy of fatigue detection, Zhu T et al. [5] proposed a novel real-time comprehensive fatigue detection algorithm by combining facial video sequence recognition technology and deep convolutional networks. The experimental findings indicated that the algorithm had significant advantages in the speed and efficiency of detecting facial fatigue in personnel. Goumopoulos C et al. [6] believed that existing wearable human fatigue detection devices required massive sensors to be connected to the device, and the detection accuracy was greatly affected by the environment. To this end, researchers proposed a novel detection method by combining human heart rate variability feature recognition and support vector machine algorithm. The experiment findings indicated that this method had high effectiveness in detecting changes in heart rate characteristics in various situations, proving its reliability in fatigue detection. Yi Y et al. [7] believed that a single fatigue detection algorithm lacked comprehensive performance and could not adapt to multi-population detection. To this end, researchers proposed a fatigue detection model that considers multiple features of the eyes and the optimal weight distribution. The experiment findings denoted that the highest accuracy of the model for fatigue detection in multiple scenarios and groups was close to 93.5%. Zheng H et al. considering the diversity and individual variability of driving environments, driver fatigue state, and the uncertainty of key characterization factors, proposed a deep learning-based MAX-MIN driver fatigue detection algorithm. The experimental results showed that the accuracy of the method was 98.8%, the recall rate was 90.2%, and the F1 score was 94.3% [8]. Dogan S et al. tried to accurately detect fatigue using a handmade frame, and proposed a fatigue detection model that combines wavelet transform and frequency domain extraction. The experimental results showed that the fatigue classification accuracy of the model reached 99.90% respectively, proving its accurate detection of fatigue [9].

Extreme Inception (Xception) is a convolutional neural network architecture proposed by the Google research team [10]. Compared to traditional fatigue detection methods that rely solely on explicit features such as facial expressions and eye movements, Xception networks can use their powerful feature extraction capabilities to deeply explore potential features such as microscopic expression changes, eye movement trajectories, and facial muscle states, thereby achieving accurate recognition of fatigue states. To raise the accuracy of fatigue detection for sports students, Liu P et al. [11] suggested a new detection algorithm that combines Xception and skin photoelectric volume fatigue level. The experiment findings denoted that this algorithm had higher accuracy in fatigue detection for sports academic than traditional algorithms. Husain S S et al. [12] argued that using historical data to train fatigue detection models was highly controversial. Therefore, researchers proposed a novel human fatigue detection method that combines Xception networks and DCNN. The experiment outcomes denoted that the average accuracy of fatigue detection for different types of workers using this method was the highest at 93.7%. Siddiqui H U R et al. [13] believed that the use of artificial intelligence could effectively detect driver drowsiness and fatigue, which could help prevent accidents and improve driver performance. To this end, researchers proposed a novel detection algorithm using Xception networks and extreme gradient boosting algorithms. The experimental outcomes denoted that the algorithm could achieve a maximum accuracy of 94.3% in detecting human drowsiness and fatigue. Mate P et al. [14] suggested a new detection method by combining Xception network and transfer learning model to further optimize the reliability of deep learning in driver fatigue monitoring. The experimental findings indicated that the fatigue detection recall rate of this method was the highest at 95.33%, which was 3.17% higher than traditional methods. Cui J et al. [15] found that the decision-making method for human fatigue level using electroencephalogram analysis was easily affected by noise. Therefore, the researchers used Xception network to process and optimize the data analysis, and finally proposed an improved electroencephalogram human fatigue detection model. The experimental outcomes illustrated that the average detection accuracy of this method for 20 subjects was 91.27%, which was significantly improved compared to before the improvement.

In summary, although there have been a number of studies on deep learning-based air traffic controller work state recognition methods, most of these methods focus on feature extraction using standard deep convolutional neural networks, such as Xception, and do not fully consider the multimodal data characteristics of air traffic controllers and their temporal dynamics in a high-load work environment. Compared to these methods, the study introduces an air traffic controller fatigue state recognition model based on an improved Xception network by optimizing the Mini-Xception network structure, first using Deep Convolutional Neural Networks (DCNN) for feature extraction optimization, and then combining with Deep Neural Networks (DNN) for feature fusion to enhance the classification stability and accuracy of the model. The innovation of the study is that by improving Mini-Xception network and combining it with dynamic time-series data processing module, a multi-task learning framework is proposed, which is able to process facial expression, eye fatigue and other physiological features at the same time, and realize the joint recognition of work state and emotional changes. In addition, the research has optimized the fatigue detection in the eye region by adopting the fusion feature extraction technique of DCNN and DNN, which makes the model’s real-time performance and accuracy in complex environments significantly improved, and solves the limitations of the traditional methods in dealing with the changes in the head posture and the unstable lighting conditions.

## 3. Methods and materials

In response to the challenges in fatigue detection for air traffic controllers, such as insufficient detection accuracy and poor real-time performance, this study first obtains data from a standard facial image dataset. Based on Mini-Xception, the convolution operation, activation function, optimizer, and loss function are sequentially replaced and optimized to propose a facial fatigue detection model. Secondly, based on the facial recognition model, the Mini-Xception network is further optimized to focus on fatigue recognition in the eye area. DCNN and DNN are introduced for fusion feature processing, and the high-level feature extraction of DCNN and deep feature integration of DNN are combined to strengthen the classification stability and accuracy of the model. Finally, a new model for detecting eye fatigue based on improved Mini-Xception is proposed.

### 3.1. Construction of facial fatigue detection model based on xception network

**Fig 1:**
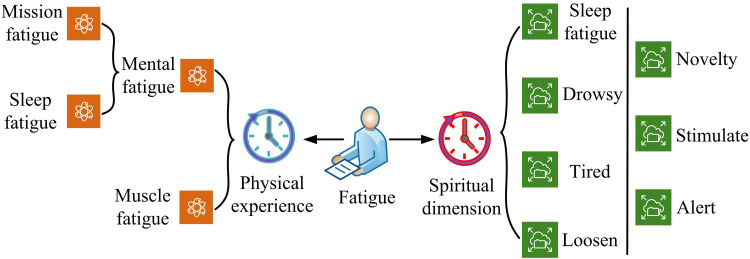
Schematic diagram of fatigue level classification.

Human fatigue is a complex physiological and psychological state, caused by a combination of factors such as sustained physical or mental activity, emotional stress, lack of rest, or environmental factors that result in energy expenditure exceeding recovery capacity, leading to a series of physiological, psychological, and behavioral reactions [16]. Fatigue is not only manifested as physical weakness, fatigue, and exhaustion, but also accompanied by cognitive decline such as lack of concentration, delayed reactions, and decreased judgment. The classification of fatigue levels is shown in Fig 1 [17].

**Fig 2:**
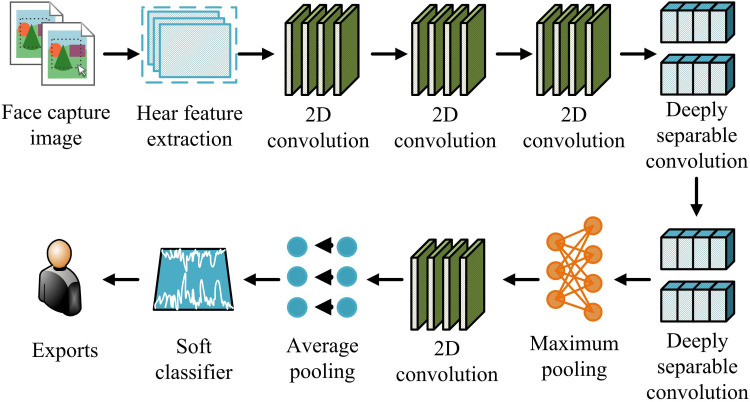
Schematic diagram of Mini-Xception’s network structure.

In Fig 1, fatigue is divided into two main categories: the physical experience and the mental dimension. Among them, the physical experience includes task fatigue, sleep fatigue, and muscle fatigue; while the mental level contains states such as relaxation, tiredness, lethargy, and sleep. Although it can be visually perceived with the naked eye, traditional recognition techniques cannot perform adaptive recognition and classification due to the expressionless and unconscious state generated by the face [18–19]. For this purpose, the Xception network is introduced for the classification and recognition of human fatigue facial features. Compared to other networks, the Xception network has a unique architecture of depthwise separable convolutions, which enables the model to have higher accuracy and efficiency in capturing facial micro expressions and fatigue features [20–21]. Mini-Reception is a unique simplified Xception network, whose network structure is shown in Fig 2.

**Fig 3:**
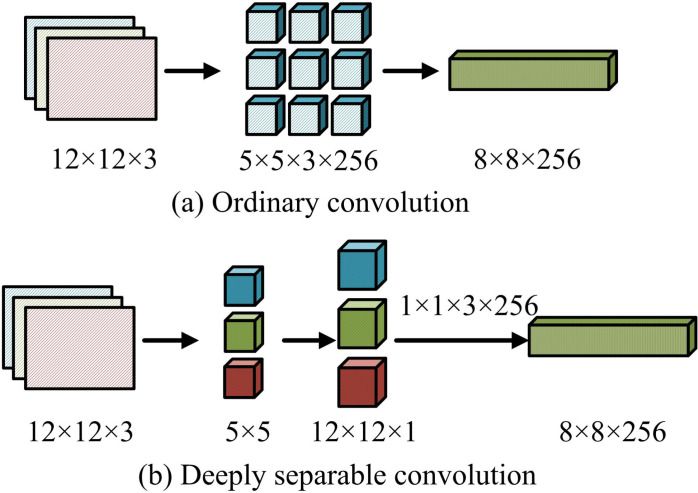
Schematic comparison of normal convolution and depth-separable convolution.

In Fig 2, the Mini-Xception network structure inherits the core idea of the Xception network. Firstly, the target area is located using a facial detector based on Hear features, and then multiple 2D convolution operations are performed sequentially. Next, the core of the network includes multiple depth separable 2D convolution modules, which process spatial and channel information separately by decomposing traditional convolution operations. Each separable convolution module is followed by a max pooling layer to lessen the size of the feature map while preserving the most significant feature information [22]. At the end of the network, there is a Fully Connected Layer (FCL) that classifies the extracted features using the Softmax activation function and ultimately outputs the prediction results [23–24]. FCLs are usually used in the final stage of the network to map the high-dimensional features extracted from the convolutional layers to the final classification result. FCLs enable the network to synthesize multiple features for the final decision by connecting all the neurons in the previous layer to each neuron in the current layer. The comparison diagram between the ordinary module and the separable convolution module is shown in Fig 3.

Fig 3 (a) shows a regular convolution, while Fig 3 (b) shows a depthwise separable convolution. In Fig 3, in ordinary convolution, the initial detection image of 12 × 12 × 3 is input, and after 5 × 5 × 3 × 256 convolution kernels and 1920 parameter operations, the output detection image size is 8 × 8 × 256. In depthwise separable convolution, the initial input is a detection image of 12 × 12 × 3 size, and a 5 × 5 pixel convolution kernel is used to perform point by point convolution on three 12 × 12 × 1 input images to obtain three features of 1 × 1 × 3 × 256 pixels. The final output is the same result image of 8 × 8 × 256, but the number of parameters involved during this period is reduced to 1200. During this period, the calculation expression for depthwise separable convolution is shown in equation (1).


$$Z_{i,j}^{k}=\sum_{m=-r}^{r}\sum_{n=-r}^{n}X_{i+m,j+n}^{k}\cdot W_{m,n}^{k}$$ (1)

In equation (1), $Z_{i,j}^{k}$ represents the output feature map of position (i,j) on k channels; $X_{i+m,j+n}^{k}$ represents the pixel values of the output feature map at position (i+m,j+n); $W_{m,n}^{k}$ denotes the convolutional kernel weights on k channels. The calculation expression for point wise convolution is shown in equation (2).


$$Y_{i,j}^{c}=\sum_{k=1}^{K}Z_{i,j}^{k}\cdot W_{1,1}^{k,c}$$ (2)

In equation (2), $Y_{i,j}^{c}$ denotes the value of the output feature map at position (i,j) on channel c; $W_{1,1}^{k,c}$ represents the weight before the k th input channel and the c th output channel. In addition, as the depth of network models continues to increase, the original activation function, namely the Softmax activation function, becomes more difficult to process nonlinear composite data. Therefore, the study replaced the Softmax activation function with a Rectified Linear Unit (ReLU) activation function. The calculation method at this time is shown in equation (3).


f(x)=max(0,x) (3)

In equation (3), x represents the input and output parameters. x stands for ReLU activation function. When x is less than 0, the output result is also 0, and the Mini-Xception network is sparser, thereby accelerating computational efficiency. When x is greater than 0, the output result is a constant value, thus avoiding the problem of gradient explosion. During network training, common objective optimization problems can be attributed to non-convex problems, where gradient descent can get stuck in local optima during parameter training, resulting in unstable training results [25–26]. To this end, the study introduces the more mainstream Adam optimizer, which combines the advantages of momentum optimizer and root mean square propagation optimizer, with fast convergence speed and strong adaptability, especially suitable for dealing with sparse gradient problems and noisy data [27–28]. The calculation method of Adam optimizer is shown in equation (4).


$$\theta_{t+1}=\theta_{t}-\frac{\eta }{\sqrt{{\hat{v}}_{t}+\varepsilon }}\cdot {\hat{q}}_{t}$$ (4)

In equation (4), η represents the learning rate; ${\hat{v}}_{t}$ and ${\hat{q}}_{t}$ both represent the average exponential decay; $\theta_{t}$ represents the parameters after t -th iterations. At this point, the loss function of the Mini-Xception network is determined by the difference between the true value and the predicted value, as denoted in equation (5).


$$\begin{array}{c} \left\{ \begin{array}{c} @lloss=-\sum_{i=1}^{n}y_{i}\log{\hat{y}}_{i}\\ \frac{\varsigma loss}{\varsigma y_{i}}=-\sum_{i=1}^{n}\frac{{\hat{y}}_{i}}{y_{i}} \end{array}\right. \end{array}$$ (5)

**Fig 4:**
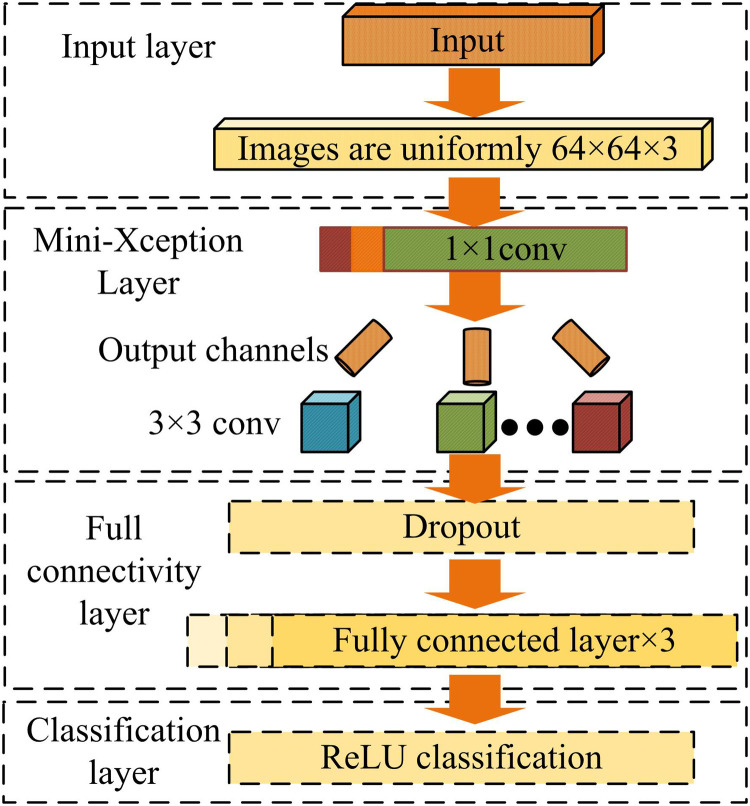
Model structure for facial fatigue expression recognition in the face.

In equation (5), $y_{i}$ and ${\hat{y}}_{i}$ output the old and new values, respectively; ς represents a variable. At this point, the improved Mini-Xception network uses ReLU activation function and Adam optimizer, and determines the loss function variable based on the difference between the true and the predicted values. A facial fatigue expression recognition model is proposed based on the improvement of the Mini-Xception network mentioned above. The structure of the model is denoted in Fig 4.

In Fig 4, the entire facial fatigue expression recognition model mainly consists of four parts, namely the input layer, Mini-Xception module, FCL, and classification layer. The input layer receives the original input facial image of the model, with a size of 64 × 64 × 3, representing a three channel color image of size 64 × 64. The core of the model is the Mini-Xception module, which uses a set of depthwise separable convolutional layers to extract high-order facial features. Specifically, the Mini-Xception module performs channel dimensionality reduction by first performing a 1 × 11 convolution operation, then dividing its output into several branches, each branch performing an independent 3 × 3 convolution operation, and finally convolving the outputs of these branches point by point. The FCL consists of multiple FCLs and a Dropout layer. The Dropout layer is utilized to randomly discard the output of some neurons to prevent over-fitting of the model. The output of the model goes through the ReLU classification layer to classify the final extracted features.

### 3.2. Construction of an eye fatigue recognition model based on improved xception

**Fig 5:**
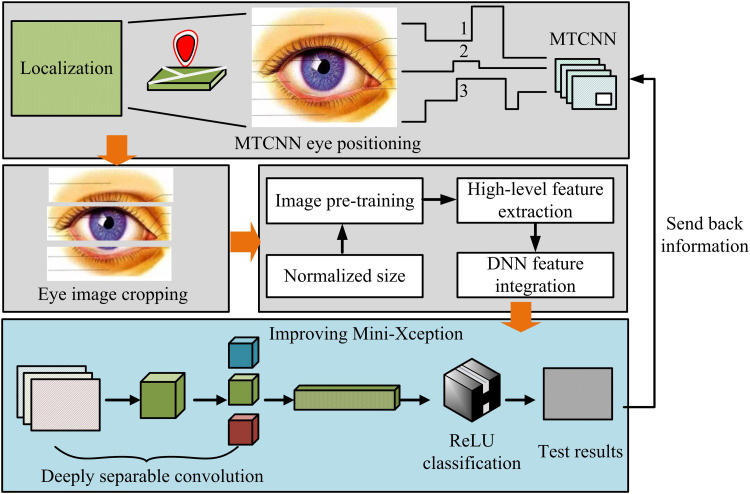
Novel human eye fatigue detection model structure.

After constructing the Mini-Xception facial fatigue expression recognition model with improved structure, the study found that compared to recognizing the entire facial fatigue expression, fatigue detection for human eyes is more convincing. Eye features are the earliest features used by scientists to detect fatigue, including blink amplitude, frequency, eye closure time, and head posture, which are particularly significant in fatigue states [29–30]. Therefore, focusing on fatigue detection in the eye area can not only improve the accuracy of detection, but also better capture early signs of fatigue, providing scientific basis for timely response measures. In addition, the eye area is more concentrated and has prominent features compared to other parts of the face, reducing redundant information in the feature extraction process and helping to raise the computational efficiency and real-time performance of the model [31]. To this end, the study further improves the structure of the Xception network, focusing on feature extraction and fatigue recognition in the eye area, and proposes a novel eye fatigue detection model. The structure of the model is denoted in Fig 5.

In Fig 5, the entire novel human eye fatigue detection model consists of six parts, i.e., eye region localization, eye image cropping, pre-training, Deep Convolutional Neural Networks and Deep Neural Networks (DCNN-DNN) fusion feature processing, Mini-Xception recognition, and final output prediction. First, the MTCNN module is used for eye region localization to ensure accurate capture of the target region. Subsequently, the localized eye images are cropped and normalized to unify the input dimensions. Then, the model utilizes large-scale image data for feature extraction in the pre-training stage and combines DCNNs and DNNs for high-level feature fusion. Finally, the improved Mini-Xception network utilizes deep separable convolution to achieve fatigue state classification and outputs “fatigue” or “non-fatigue” judgment results. In practical control work, due to the frequent scanning of radar screens by controllers, head rotation may cause one eye to be obstructed, thereby affecting the accuracy of fatigue detection [32]. Therefore, when the head rotation angle is too large, the model should prioritize detecting unobstructed eyes and select the single eye with higher detection confidence when both eyes are unobstructed. The monocular localization calculation method of MTCNN is shown in equation (6).


$$\left\{ \begin{array}{c} \left(x_{L},y_{L})=FaceDetector(I,left-eye\right)\\ \left(x_{R},y_{R})=FaceDetector(I,right-eye\right) \end{array}\right.$$ (6)

In equation (6), I represents the facial output image; FaceDetector stands for MTCNN detection algorithm; $\left(x_{L},y_{L}\right)$ and $\left(x_{R},y_{R}\right)$ represent the position coordinates of the left and right eyes, respectively. During this period, the angle of head rotation can be estimated by the difference in position between the left and right eyes. Assuming the horizontal distance between the left and right eyes is Δx, the estimation expression for the head rotation angle is shown in equation (7).


$$\omega =\arctan(\frac{\left|x_{R}-x_{L}\right|}{d})$$ (7)

In equation (7), ω represents the angle of head rotation; $\left|x_{R}-x_{L}\right|$, Δx stands for the horizontal distance between the left and right eyes; d represents the reference distance, usually the vertical distance from the eyes to the camera. When the head rotation angle ω is greater than the threshold, the screening mechanism at this time is shown in equation (8).


$$S\text{e}lectedEye=\left\{ \begin{array}{c} Left-eye,ifx_{L}>x_{R}andConf(L)\geq Conf(R)\\ Reft-eye,ifx_{R}>x_{L}andConf(R)\geq Conf(L) \end{array}\right.$$ (8)

In equation (8), SelectedEye represents the selected eye; Conf(L) and Conf(R) represent left eye confidence and right eye confidence, respectively. When the head rotation angle is less than the threshold, the eyes with higher confidence are selected for detection, as shown in equation (9).


$$S\text{e}lectedEye=\left\{ \begin{array}{c} Left-eye,ifConf(L)\geq Conf(R)\\ Reft-eye,ifConf(R)>Conf(L) \end{array}\right.$$ (9)

**Fig 6:**
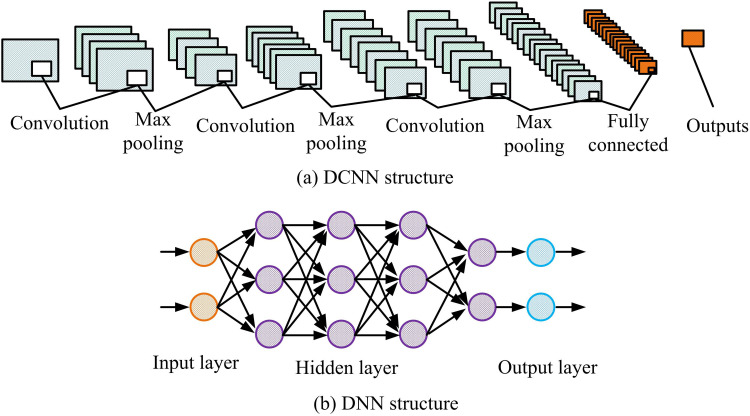
Network architecture of DCNN and DNN.

By combining equations (6) to (9) with this screening mechanism, the model can effectively handle the problem of eye occlusion caused by head rotation and select the most suitable eye for fatigue state detection. In addition, the network structures of DCNN and DNN are denoted in Fig 6 [33].

**Fig 7:**
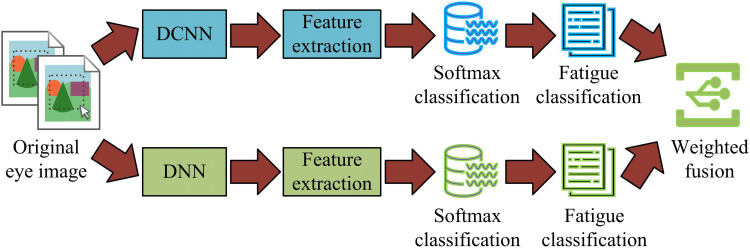
Schematic diagram of DCNN-DNN fusion feature processing.

Figs 6 (a) and (b) are schematic diagrams of the network structure of DCNN and DNN, respectively. In DCNN, the model gradually extracts high-level features through multi-layer convolution and pooling, while reducing the spatial size of the feature map, and finally completing the mapping of features to the output classification through the fully connected layer. In DNN, the model uses multi-layer hidden layers to process the input features step by step, and then completes the extraction and combination of non-linear features by combining the fully connected and activation functions, and then maps them to the output layer to complete the classification. Overall, both models have their own advantages in feature extraction and are suitable for different types and complexity of data. By combining the feature extraction capabilities of DCNN and DNN, the classification accuracy and robustness of the model can be further improved. The research combines the two and proposes DCNN-DNN fusion feature processing to improve the fatigue feature recognition performance of Mini-Xception. The schematic diagram of DCNN-DNN is shown in Fig 7.

In Fig 7, the original eye image is first obtained, and the image is simultaneously input to both the DCNN model and the DNN model. The DCNN model and DNN model independently extract features and perform preliminary classification judgments, and obtain corresponding fatigue state judgment results through the Softmax layer. Next, the results of these two paths are fused through kernel based feature fusion. The classification results of DCNN and DNN are comprehensively considered, and a weight-based fusion decision method is used to obtain the final fatigue detection result. The feature extraction and classification of DCNN and DNN are denoted in equation (10).


$$\left\{ \begin{array}{c} P_{DCNN}=Softmax(W_{DCNN}\cdot f_{DCNN}+b_{DCNN})\\ P_{DNN}=Softmax(W_{DNN}\cdot f_{DNN}+b_{DNN}) \end{array}\right.$$ (10)

In equation (10), $f_{DCNN}$ and $f_{DNN}$ represent the feature vectors extracted by DCNN and DNN, respectively; $W_{DCNN}$ and $W_{DNN}$ represent the classification weight matrices of DCNN and DNN, respectively; $b_{DCNN}$ and $b_{DNN}$ represent the bias vectors of DCNN and DNN, respectively; $P_{DCNN}$ and $P_{DNN}$ represent the classification probability distributions obtained by the Softmax layer in DCNN and DNN, respectively. The calculation expression for fusing classification features is shown in equation (11).


$$f_{fusion}=\alpha \cdot f_{DCNN}+\beta \cdot f_{DNN}$$ (11)

In equation (11), $f_{fusion}$ represents the fused feature vector; α and β both represent weight coefficients. The fused feature vector $f_{fusion}$ is input into the optimized Mini-Xception model for final fatigue state classification, as shown in equation (12).

**Fig 8:**
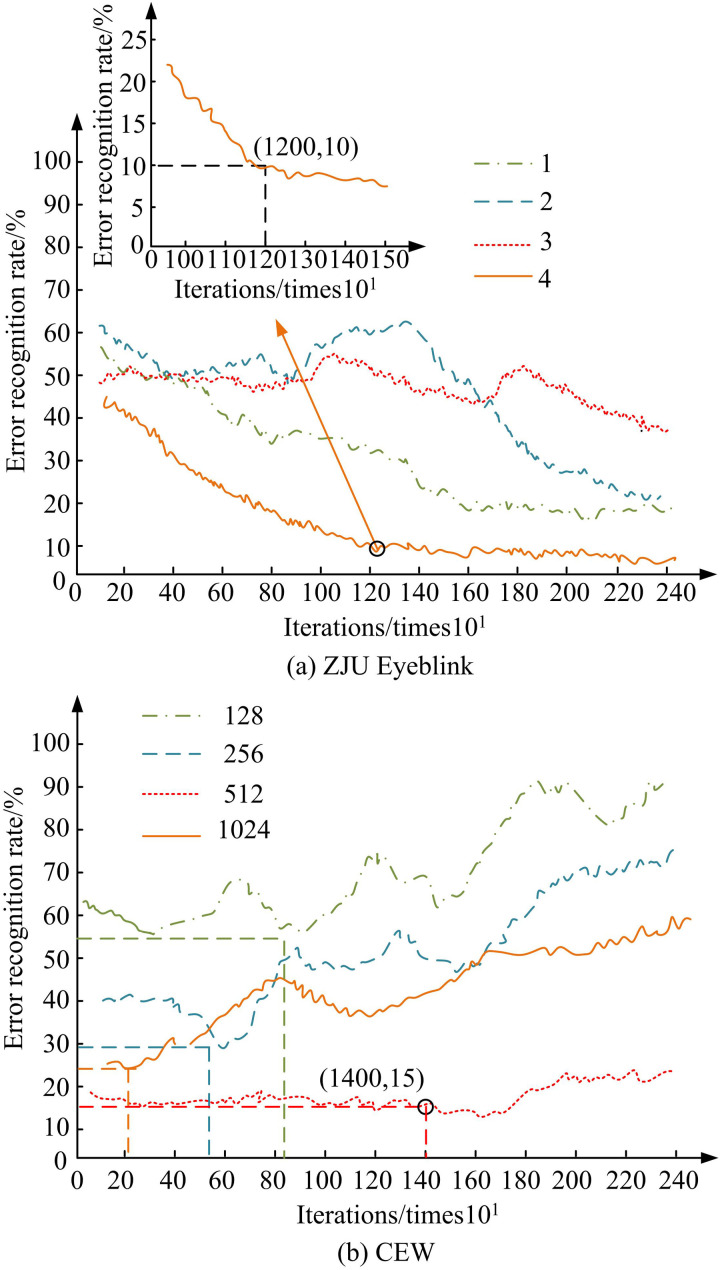
Model performance test results with different parameter settings.

**Fig 9:**
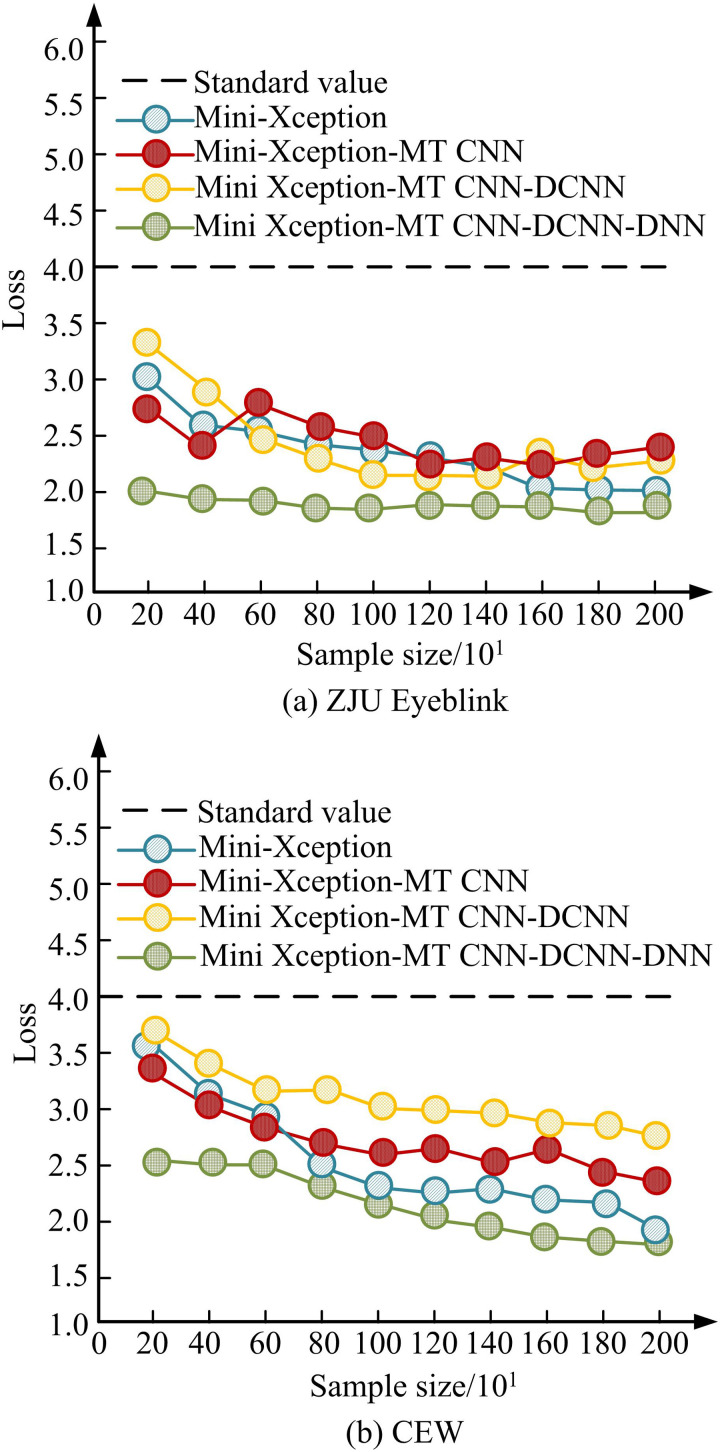
Improved ablation test results for the Mini-Xception network.

**Fig 10:**
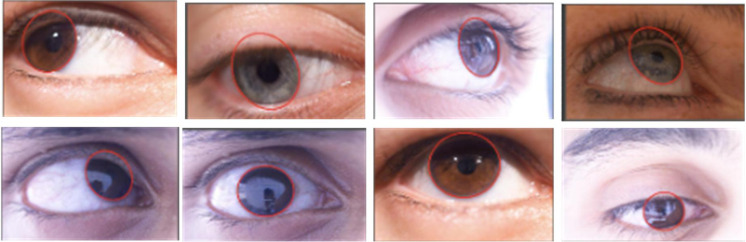
Test image display.

**Fig 11:**
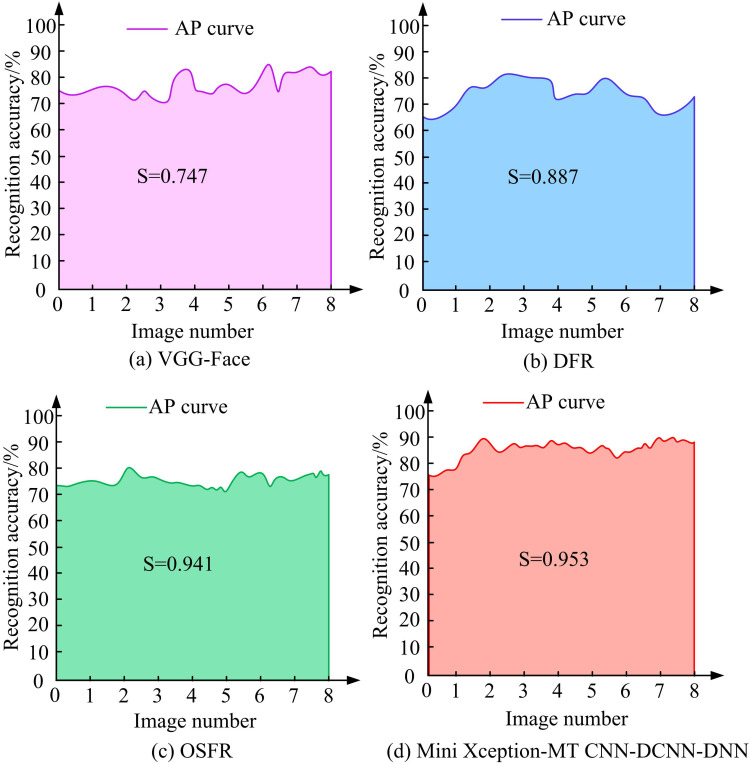
Test AP results of different models for 8 graphs.

**Fig 12:**
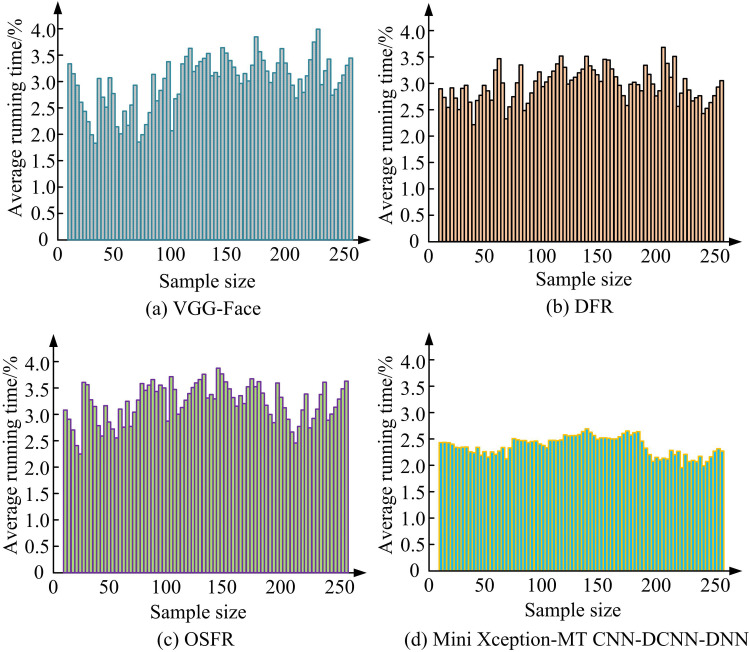
Average runtime test results for different models.


$$P_{final}=Softmax(W_{MX}\cdot f_{fusion}+b_{MX})$$ (12)

In equation (12), $W_{MX}$ represents the classification weight matrix of Mini-Xception; $b_{MX}$ represents the bias vector of the Mini-Xception model; $P_{final}$ represents the final classification result of the Mini-Xception model, which is the probability distribution of different fatigue states.

## 4. Results and discussion

In order to test the performance effect of the newly proposed cyber security perception model, the study first builds a suitable experimental environment. Several datasets were selected for the experiments, and the performance of the model in terms of accuracy, precision, recall, F1 value, AUC, and other metrics were evaluated by comparing it with other state-of-the-art models. Additionally, ablation tests were conducted to gradually introduce different modules to explore the contribution of each module to the overall model performance. In addition, fatigue detection was performed with real 8 categories of eye images, while advanced algorithmic models were introduced for comparison to validate the real effectiveness of the proposed model in the study.

### 4.1. Performance testing of fatigue identification model

The study uses the Zhejiang University Eyeblink Database (ZJU Eyeblink) and the Closed Eyes in the Wild Dataset (CEW) in natural environments as test data sources. ZJU Eyeblink is an eye movement dataset created by Zhejiang University that contains eye images from 100 subjects with a total sample size of 10,000 images. The categories of the dataset include “fatigue” and “non-fatigue”, where the sample size of each category is 5,000 images. The distribution of categories in this dataset is well balanced, with no significant imbalance, while the CEW dataset contains ocular images from 150 subjects, with a total sample size of 15,000 images. Similar to the ZJU dataset, the CEW dataset is divided into two categories, “fatigue” and “non-fatigue”. The sample size for each category is 7,500 images, and the distribution between categories is also balanced. The above data sources are pre-processed, including data augmentation, data extraction, and data partitioning. The specific equipment and parameters for the experiment are denoted in Table 1.

**Table 1:** Experimental environment and parameters.

Item	Parameter
Programming Language	Python 3.7.13
CPU	Intel Core i7 3.6Hz
GPU	Nvidia GeForce GTX TITAN X
Memory	32G
Backbone	CNN
Deep learning framework	Pytorch 1.13.1
Neural network construction method	Keras
Batch size	16
Iiterations	100
Loss function ratio	1:01
Initial learning rate	0

Before starting the experiments, the study carried out the necessary parameter settings and optimization of the model.Mini-Xception network was used with ReLU activation function, Adam optimizer with a learning rate of 0.001 and cross-entropy loss function. For data preprocessing, data enhancement techniques such as image rotation, flipping and illumination changes were used. For hyper-parameter tuning, the fully connected layer is set to 4 layers, Dropout ratio is 0.5, batch size is 16, and the number of iterations is 100. After setting up the experimental environment, the study first determines the two hyperparameters of the improved Mini-Xception model to achieve optimal model performance. The FCL plays a key role in data classification, with its number set at levels 1, 2, 3, and 4, while Dropout uses regularization to avoid over-fitting caused by too many layers in the model, with values of 128, 256, 512, and 1024. The test findings are denoted in Fig 8.

Fig 8 (a) showcases the performance test outcomes of the model with different FCL data settings on the ZJU Eyeblink dataset. Fig 8 (b) showcases the performance test outcomes of the model with different Dropout values set on the CEW dataset. In Fig 8 (a), as the continuous increase of iteration times and the number of FCLs, there is a significant decrease in the model’s eye fatigue recognition error rate. When the number of FCLs is 4, the minimum model detection error can reach 10%, and the number of iterations is 1200. From Fig 8 (b), the Dropout value is similar to the data performance of the FCL, but there is a certain fluctuation in the model performance when the Dropout value is 1024. Relatively speaking, the fatigue detection data of the model is more stable when Dropout was 512, and the lowest fatigue detection error at this time can reach 15%. It is thus illustrated that the gap between the training error and the validation error of the model is significantly reduced by introducing L2 regularization and data augmentation on top of the Dropout regularization, indicating that the over-fitting phenomenon is effectively mitigated. The performance of the final model on the validation set is more stable, further proving the effectiveness of the proposed optimization strategy. Therefore, the study determines a Mini Xception-MT CNN-DCNN-DNN model with an FCL as 4 layers and a Dropout value of 512 for subsequent testing. Ablation testing is performed on the improved Mini-Xception model to investigate the performance impact of each module on the overall algorithm, such as Mini-Xception, Mini-Xception-MT CNN, Mini-Xception MT CNN-DCNN, and Mini-Xception-MT CNN-DCNN-DNN. The test results of the accuracy of facial eye fatigue recognition for four modules as a function of iteration times are shown in Fig 9.

Fig 9 (a) showcases the ablation test outcomes of the improved Mini-Xception in the ZJU Eyeblink dataset, and Fig 9 (b) showcases the ablation test outcomes of the improved Mini-Xception in the CEW dataset. In Fig 9 (a), with the continuous increase of the amount of test samples, the lowest value of the final model loss function test result after sequentially introducing MT-CNN, DCNN, and DNN into Mini-Xception can reach 1.8. In Fig 9 (b), after improving Mini-Xception, the model detection performance shows a linear improvement. The mini loss value for the final improved Mini-Xception model is 2.0. As a result, it can be shown that the introduction of the modules in Mini-Xception, despite the relatively small performance improvement at the macro level, further analysis reveals that the fusion model exhibits significant advantages when dealing with complex samples and multidimensional features. In the ZJU Eyeblink dataset, the fusion model significantly outperforms Mini-Xception in recognizing rare and anomalous samples, which is reflected in the significant decrease in the Loss value when the number of samples is small, which is about 0.8. While in the CEW dataset, due to the large variations in sample illumination and poses, the fusion model is able to better capture these complex features, and thus, at the early stage of training that exhibits lower Loss values. In addition, the fusion model enhances the ability to process multimodal information by introducing multi-task learning and feature fusion techniques, which is still noteworthy in terms of robustness and stability, even in the case of large sample sizes. The study introduces advanced detection models of the same type for comparison, such as Inception-ResNet, Inception Version 4 (Inception-V4), and Xception-DeepLab. The testing was conducted using Precision (P), Recall (R), F1 score (F1), and Area Under the Curve (AUC) as indicators, and the test outcomes are denoted in Table 2.

**Table 2:** Indicator test results for the same type of model.

Dat set	Model	Accuracy/%	P/%	R/%	F1/%	AUC/%	Complexity/%	Average time/s
ZJU Eyeblink	Inception-ResNet	86.44	87.66	85.28	86.47	91.37	70.76	0.35
	Inception-V4	87.65	88.97	87.63	88.31	90.22	72.18	0.32
	Xception-DeepLab	89.71	90.57	87.54	89.06	92.54	85.69	0.45
	Mini Xception-MT CNN-DCNN-DNN	91.27	93.41	90.28	91.85	95.73	60.44	0.28
CEW	Inception-ResNet	88.87	89.78	84.76	87.27	90.69	70.88	0.33
	Inception-V4	89.36	86.52	86.85	86.69	92.66	72.36	0.31
	Xception-DeepLab	90.12	90.43	89.79	90.11	91.86	85.58	0.44
	Mini Xception-MT CNN-DCNN-DNN	92.73	94.36	91.68	93.02	95.21	61.31	0.29

From Table 2, the accuracy of the Institute’s proposed Mini Xception-MT CNN-DCNN-DNN model in the ZJU Eyeblink dataset is 91.27%, which is an improvement of 1.56% compared to Xception-DeepLab, and in terms of precision, recall, F1 value, and AUC, respectively, it is 93.41%, 90.28%, 91.85% and 95.73%, which are all significantly better than Xception-DeepLab, especially in the AUC value, which is improved by 3.19%. In the CEW dataset, the accuracy of the proposed model is 92.73% and the AUC value is 95.21%, which are improved by 2.61% and 3.35% compared with Xception-DeepLab, respectively. In terms of computational complexity, Mini Xception-MT CNN-DCNN-DNN has a complexity of 60.44% and 61.31%, which is significantly lower than that of Xception-DeepLab’s 85.69% and 85.58%, respectively. In addition, in terms of average elapsed time, Mini Xception-MT CNN-DCNN-DNN also exhibits shorter processing times of 0.28 and 0.29 seconds, which are significantly more efficient than Xception-DeepLab’s 0.45 and 0.44 seconds, respectively. Taken together, the proposed model outperforms the other models in all indicators, showing stronger performance and computational efficiency, and is especially suitable for practical application scenarios that require higher processing speed and accuracy.

### 4.2. Fatigue identification model simulation testing

To more accurately verify the detection results of the proposed model under the fatigue state of traffic controllers, 8 eye images from the CEW public dataset are randomly selected for testing. These images are all publicly available data to ensure there are no privacy or copyright issues, and they cover different lighting conditions and head postures to simulate complex situations in actual work environments. By conducting fatigue detection on these images, research can evaluate the robustness and accuracy of the model in diverse scenarios. The test image is denoted in Fig 10.

From Fig 10, various types of eye detection images are captured under different lighting conditions and postures to achieve the effect of simulating real working conditions. A 5-fold cross-validation is performed on the above 8 categories of randomly selected images and the results are shown in Table 3.

**Table 3:** Fifty-fold cross-validation results for randomized test images.

Fold	Training set (Combination of 4 subsets)	Test set (1 subset)	F1 score (%)
Fold 1	Image 1, Image 2, Image 3, Image 4	Image 5	91.24
Fold 2	Image 1, Image 2, Image 5, Image 6	Image 3	90.56
Fold 3	Image 1, Image 3, Image 6, Image 7	Image 4	92.14
Fold 4	Image 1, Image 4, Image 5, Image 7	Image 2	89.88
Fold 5	Image 2, Image 3, Image 4, Image 8	Image 1	93.12

From Table 3, the model’s F1 score in the five-fold cross-validation is stable, and the performance difference between different subsets in the test set is small. The F1 scores for each fold range from 89.80% to 93.12%, indicating that the model has strong generalization ability. The average F1 score is 91.36% in all the five-fold tests, showing that the model performs well in the classification task of eight classes of randomly selected images, effectively balancing precision and recall, and showing no obvious over-fitting. The study introduces advanced facial expression detection models, such as Visual Geometry Group Face (VGG Face), Deep Face Recognition (DFR), and Open Source Face Recognition (OSFR). DFR is based on DCNNs and is capable of extracting rich facial features from face images for face recognition. In air traffic controller work state recognition, the DFR model is used to analyze the controller’s facial expression and emotional changes to help identify his current work state. OSFR open source face recognition models are a class of publicly released tools or frameworks that allow developers to use and modify the models for specific applications. OSFR is usually based on deep learning algorithms, and is able to efficiently perform face detection and face recognition tasks OSFR is usually based on deep learning algorithms that can efficiently perform face detection and face recognition tasks, adapting to a wide range of real-world applications. The Average Precision (AP) was used as the indicator for testing, and the test findings are denoted in Fig 11.

Figs 11 (a), (b), (c), and (d) showcase the AP detection outcomes of the VGG-Face, DFR, OSFR models, and the proposed model on 8 eye images, respectively. According to Fig 11, after fatigue detection on eight different eye images, the area under the AP curve of the VGG-Face model is maximized at 0.747, the area under the AP curve of the DFR model is maximized at 0.887, the area under the AP curve of the OSFR model is maximized at 0.941, and the area under the AP curve of the research-proposed model is maximized at 0.953, which demonstrates significant improvement. Compared with the other models, the AP value of the proposed model is significantly higher, and its detection results are more stable and reliable, especially in the face of different eye images, which can maintain consistency. Further analyzed, although the VGG-Face and DFR models are able to achieve better detection results on certain images, their AP curves fluctuate more and lack certain robustness. On the other hand, studying the proposed model can provide more accurate detection results under different image conditions, and its improved Mini-Xception network effectively reduces the number of parameters through depth-separable convolution, which improves the computational speed and reduces the risk of overfitting while ensuring high efficiency. This makes the proposed model of the study still able to realize high-precision fatigue detection under complex environments, especially under large differences in image quality and attitude, and has strong application prospects and practical value. Continuing with the average running time as the indicator, the test results are shown in Fig 12.

Figs 12 (a), (b), (c), and (d) showcase the average running time test findings of the VGG-Face, DFR, OSFR models, and the proposed model, respectively. In Fig 12, the test results of the VGG-Face model has significant fluctuations, with the lowest average running time being 1.9 seconds. Although the test results of DFR and OSFR models have small fluctuations, their respective data are generally biased, with the highest being close to 4 seconds. Relatively speaking, the proposed model takes an average of 2.3 seconds for detecting eye fatigue, and in the later stage of testing with a large sample size, the model also shows relatively stable testing time data. To prove the efficacy of the proposed model in complex data backgrounds, different numbers of samples are tested, and the test findings are indicated in Table 4.

**Table 4:** Accuracy test results of model detection with different data volumes.

Number of images	VGG-Face	DFR	OSFR	The method of Zhang J et al.	The method of Zhang Z et al.	The method of Zhu T et al.	The approach of Goumopoulos C et al.	Our model
20	0.92	0.83	0.82	0.85	0.88	0.89	0.87	0.93
40	0.88	0.86	0.89	0.86	0.87	0.92	0.88	0.92
60	0.83	0.81	0.87	0.84	0.86	0.85	0.89	0.91
80	0.84	0.83	0.84	0.82	0.83	0.86	0.84	0.89
100	0.91	0.89	0.84	0.88	0.91	0.91	0.91	0.93
120	0.82	0.84	0.81	0.85	0.84	0.87	0.83	0.88
140	0.76	0.84	0.82	0.85	0.82	0.84	0.85	0.88
160	0.84	0.88	0.89	0.87	0.85	0.88	0.87	0.93
180	0.91	0.87	0.90	0.88	0.91	0.89	0.91	0.95
200	0.89	0.86	0.88	0.85	0.86	0.84	0.88	0.89
220	0.91	0.82	0.91	0.89	0.87	0.91	0.91	0.94
240	0.93	0.86	0.88	0.87	0.88	0.85	0.83	0.89
260	0.86	0.79	0.85	0.83	0.8	0.82	0.84	0.88
280	0.84	0.82	0.81	0.83	0.82	0.85	0.86	0.87
300	0.83	0.79	0.82	0.81	0.82	0.79	0.83	0.89
Average value	0.86	0.84	0.86	0.85	0.85	0.87	0.88	0.91

According to Table 4, the detection accuracy of the proposed Mini Xception-MT CNN-DCNN-DNN model consistently performs well under different data volumes, especially reaching 0.89 under the data volume of 300 images, which is a clear advantage over other models. In the case of small data volume (e.g., 20 images), the accuracy of Mini Xception-MT CNN-DCNN-DNN is 0.93, which is significantly better than that of VGG-Face’s 0.92 and the other methods, and the model’s performance is always high and stable with the increase of data volume. When the data volume reaches 200 sheets, the accuracy of Mini Xception-MT CNN-DCNN-DNN is 0.89, which is slightly decreased but still better than most other methods, especially VGG-Face’s 0.89 and DFR’s 0.86.Overall, the Mini Xception-MT CNN-DCNN-DNN model in all test data sizes shows strong detection ability, and its accuracy steadily improves with the increase of data size, reflecting the robustness and superiority of the model on data sets of different sizes.

## Conclusion

The study proposes an air traffic controller fatigue recognition model based on improved Xception network, aiming to address the shortcomings of existing methods in detection accuracy and real-time performance. By optimizing the convolution operation, activation function, optimizer, and loss function of Mini-Xception network, and combining multiple feature fusion techniques, including MTCNN, DCNN,and DNN, the proposed model performs well in several performance metrics. The experimental results show that the model detection error can be as low as 10% when the FCL is 4 layers, and the detection data is more stable with an error as low as 15% when the value of Dropout is 512. In the ablation test, the improvement of each module effectively reduces the Loss function value, which proves the effectiveness of the optimization measures. Compared with the same type of model, the proposed model has a P value of 94.36%, an R value of 91.68%, an F1 score of 93.02%, and an AUC value of 95.21%, which are significant improvements in all of the above aspects, especially in the AUC value of 5% compared with the Inception-V4 model. In the eye image simulation test, the area under the AP curve of the model is 0.953, and the detection time is also significantly shortened, with an average detection time of 2.3 seconds, a detection accuracy of 95%, and a mean fatigue detection accuracy of 91%.

## Limitation futurework

However, there are some limitations to this study. First, the dataset used is more limited, and there is still room for improvement, especially in terms of diversity and size. Second, although the improved Mini-Xception network has improved in computational efficiency, the performance of the model may still be somewhat affected under some extreme conditions (e.g., when the image quality is low or the angle changes are large). Future research can extend more diverse datasets, enhance the generalization ability of the model, and further improve the robustness and real-time performance of the model by introducing methods such as self-supervised learning. In addition, deeper feature fusion strategies can be explored to enhance the performance of the model in practical applications, especially in areas such as human eye fatigue detection.

## Supporting information

S1 file Minimal Data Set Definition. (DOCX)
