# Supplementary material for: Air traffic controller work state recognition based on improved xception network
Source: PLoS One. 2025 May 7;20(5):e0322404. doi: 10.1371/journal.pone.0322404 (PMC12057947; doi:10.1371/journal.pone.0322404)
Supplement: S1 file — (DOCX) [file pone.0322404.s001.docx]

**Figure 8. Model performance test results with different parameter settings**

| Iterations (times10^1^) | Error recognition rate 1 (%) | Error recognition rate 2 (%) | Error recognition Rate 3 (%) | Error recognition rate 4 (%) |
| --- | --- | --- | --- | --- |
| 10 | 80.56 | 79.89 | 80.12 | 76.99 |
| 20 | 70.24 | 69.62 | 70.05 | 67.32 |
| 30 | 61.38 | 61.26 | 62.27 | 59.21 |
| 40 | 54.22 | 54.35 | 55.49 | 52.54 |
| 50 | 47.91 | 48.12 | 49.34 | 46.12 |
| 60 | 42.81 | 43.95 | 44.18 | 40.93 |
| 70 | 38.99 | 39.93 | 39.88 | 36.93 |
| 80 | 35.46 | 36.52 | 36.35 | 33.15 |
| 90 | 32.53 | 33.74 | 33.64 | 30.56 |
| 100 | 30.08 | 31.35 | 31.15 | 28.11 |
| 110 | 28.03 | 29.46 | 29.14 | 26.02 |
| 120 | 26.22 | 27.73 | 27.28 | 24.13 |
| 130 | 24.56 | 26.14 | 25.56 | 22.42 |
| 140 | 23.11 | 24.68 | 24.23 | 20.87 |
| 150 | 21.75 | 23.29 | 22.58 | 19.46 |
| 160 | 20.47 | 22.75 | 21.28 | 18.26 |
| 170 | 19.27 | 20.83 | 20.08 | 17.06 |
| 180 | 18.14 | 19.69 | 19.71 | 16.03 |
| 190 | 17.07 | 18.66 | 18.23 | 15.09 |
| 200 | 16.06 | 17.71 | 17.15 | 14.23 |
| 210 | 15.14 | 16.82 | 16.28 | 13.44 |
| 220 | 14.18 | 15.99 | 15.55 | 12.72 |
| 230 | 13.29 | 15.22 | 14.87 | 12.06 |

**Figure 9. Improved ablation test results for the Mini-Xception network**

| Sample size (x10^1^) | Standard value | Mini-Xception | Mini-Xception-MT CNN | Mini-Xception-MT CNN-DCNN | Mini-Xception-MT CNN-DCNN-DNN |
| --- | --- | --- | --- | --- | --- |
| 1 | 5.5 | 5.4 | 5.2 | 5.1 | 5.9 |
| 2 | 5.4 | 5.3 | 5.8 | 4.9 | 4.8 |
| 3 | 5.3 | 5.1 | 4.9 | 4.7 | 4.6 |
| 4 | 5.1 | 4.9 | 4.7 | 4.5 | 4.4 |
| 5 | 5.2 | 4.8 | 4.5 | 4.3 | 4.3 |
| 6 | 4.9 | 4.7 | 4.4 | 4.2 | 4.2 |
| 7 | 4.8 | 4.6 | 4.2 | 4.1 | 4.1 |
| 8 | 4.7 | 4.5 | 4.1 | 4.5 | 4.4 |
| 9 | 4.6 | 4.3 | 4.5 | 3.9 | 3.9 |
| 10 | 4.5 | 4.1 | 3.8 | 3.8 | 3.7 |
| 15 | 4.2 | 3.9 | 3.6 | 3.7 | 3.6 |
| 20 | 4.1 | 3.7 | 3.5 | 3.6 | 3.5 |
| 30 | 3.9 | 3.6 | 3.4 | 3.5 | 3.3 |
| 40 | 3.8 | 3.4 | 3.3 | 3.4 | 3.2 |
| 50 | 3.7 | 3.2 | 3.2 | 3.3 | 3.1 |
| 60 | 3.6 | 3.1 | 3.1 | 3.2 | 3.6 |
| 70 | 3.5 | 3.3 | 3.6 | 3.1 | 2.9 |
| 80 | 3.4 | 2.9 | 2.9 | 3.4 | 2.8 |
| 100 | 3.2 | 2.8 | 2.7 | 2.9 | 2.7 |
| 150 | 3.1 | 2.7 | 2.6 | 2.8 | 2.6 |
| 200 | 3.7 | 2.6 | 2.5 | 2.7 | 2.5 |

**Figure 11. Test AP results of different models for 8 graphs**

| Image number | VGG-Face | DFR | OSFR | Mini Xception |
| --- | --- | --- | --- | --- |
| 0 | 75.36 | 84.21 | 80.12 | 88.13 |
| 1 | 74.95 | 83.91 | 79.84 | 87.95 |
| 2 | 75.12 | 84.05 | 80.23 | 88.08 |
| 3 | 75.45 | 84.11 | 80.11 | 88.21 |
| 4 | 75.32 | 84.17 | 80.06 | 88.17 |
| 5 | 75.22 | 84.01 | 80.12 | 88.14 |
| 6 | 75.17 | 83.96 | 80.18 | 88.09 |
| 7 | 75.19 | 83.97 | 80.05 | 88.11 |
| 8 | 74.94 | 84.02 | 80.08 | 88.14 |

**Figure 12. Average runtime test results for different models**

| Sample Size (x10^1^) | VGG-Face | DFR | OSFR | Mini Xception-MT CNN-DCNN-DNN |
| --- | --- | --- | --- | --- |
| 10 | 2.74 | 3.72 | 3.34 | 3.12 |
| 50 | 2.62 | 2.99 | 2.83 | 2.64 |
| 100 | 2.62 | 2.27 | 3.26 | 2.85 |
| 150 | 2.25 | 2.43 | 2.18 | 2.65 |
| 200 | 3.41 | 2.23 | 2.71 | 2.37 |
| 250 | 2.99 | 2.83 | 2.14 | 2.28 |
